# Maternally Contributed Folate Receptor 1 Is Expressed in Ovarian Follicles and Contributes to Preimplantation Development

**DOI:** 10.3389/fcell.2017.00089

**Published:** 2017-09-28

**Authors:** Trine Strandgaard, Solveig Foder, Anders Heuck, Erik Ernst, Morten S. Nielsen, Karin Lykke-Hartmann

**Affiliations:** ^1^Department of Biomedicine, Aarhus University, Aarhus, Denmark; ^2^Department of Gynaecology and Obstetrics, Aarhus University Hospital, Aarhus, Denmark; ^3^Lundbeck Foundation Research Initiative on Brain Barriers and Drug Delivery, Aarhus University, Aarhus, Denmark; ^4^Department of Clinical Medicine, Aarhus University, Aarhus, Denmark; ^5^Department of Clinical Genetics, Aarhus University Hospital, Aarhus, Denmark

**Keywords:** folate receptor 1, follicle development, preimplantation development, siRNA, blastocyst

## Abstract

Folates have been shown to play a crucial role for proper development of the embryo as folate deficiency has been associated with reduced developmental capacity such as increased risk of fetal neural tube defects and spontanous abortion. Transcripts encoding the reduced folate carrier RFC1 (SLC19A1 protein) and the high-affinity folate receptor FOLR1 are expressed in oocytes and preimplantation embryos, respectively. In this study, we observed maternally contributed FOLR1 protein during mouse and human ovarian follicle development, and 2-cell mouse embryos. In mice, FOLR1 was highly enriched in oocytes from primary, secondary and tertiary follicles, and in the surrounding granulosa cells. Interestingly, during human follicle development, we noted a high and specific presence of FOLR1 in oocytes from primary and intermediate follicles, but not in the granulosa cells. The distribution of FOLR1 in follicles was noted as membrane-enriched but also seen in the cytoplasm in oocytes and granulosa cells. In 2-cell embryos, FOLR1-eGFP fusion protein was detected as cytoplasmic and membrane-associated dense structures, resembling the distribution pattern observed in ovarian follicle development. Knock-down of *Folr1* mRNA function was accomplished by microinjection of short interference (si)RNA targeting *Folr1*, into mouse pronuclear zygotes. This revealed a reduced capacity of *Folr1* siRNA-treated embryos to develop to blastocyst compared to the siRNA-scrambled control group, indicating that maternally contributed protein and zygotic transcripts sustain embryonic development combined. In summary, maternally contributed FOLR1 protein appears to maintain ovarian functions, and contribute to preimplantation development combined with embryonically synthesized FOLR1.

## Introduction

It is well-established that folates are important for the development of the embryo. Pregnant women are advised to take a dietary supplement of 400 mg folic acid per day from 12 weeks before conception and during the first trimester of pregnancy (Hibbard, 1964; Cawley et al., 2016). Folate deficiency has been associated with increasing the risk of neural tube defects (NTD) (Detrait et al., 2005; Pitkin, 2007), early spontaneous abortion (George et al., 2002), congenital heart defects (Rosenquist et al., 2010) and orofacial clefts (Taparia et al., 2007) amongst others.

The folate receptor is highly expressed in reproductive tissues thereby providing folate to the embryo (da Costa et al., 2003). Deficiency in folate uptake can cause pregnancy-related complication such as NTD, a well-established association (Centers for Disease Control, 1991a,b; MRC Vitamin Study Research Group, 1991; Czeizel and Dudas, 1992; Cragan et al., 1995). Interestingly, the identification of folate receptor autoantibodies in in women with recurrent NTD pregnancies might mechanistically explain how the embryo is deprived of folate (Rothenberg et al., 2004; Berrocal-Zaragoza et al., 2009; Sequeira et al., 2013). Additionally, variation in folate pathway genes are associated with female infertility (Altmae et al., 2010). In line with this, postnatally acquired FR autoantibodies blocking folate transport to the brain have been associated with the infantile-onset cerebral folate deficiency syndrome (Ramaekers et al., 2005), which in a number of patients manifests as low-functioning autism with neurological deficits (Ramaekers et al., 2007). Folic acid supplementation in pregnancy may have beneficial effects on the neurodevelopment of children, such as cerebral folate deficiency syndrome and autism spectrum disorders (Desai et al., 2016), beyond its proven effect on NTDs (Blencowe et al., 2010; De-Regil et al., 2015; Gao et al., 2016).

It is proposed that folate deficiency affects gene expression by disrupting DNA methylation patterns or by inducing base substitutions, DNA breaks, gene deletions and gene amplification (Crott et al., 2008). In line with this, mutations in the enzyme methionine synthase reductase (*Mtrr*), necessary for utilization of methyl groups from the folate cycle, affect the folate metabolism and cause epigenetic instability (Padmanabhan et al., 2013). The phenotypes of the *Mtrr*-deficient mice included congenital malformations with neural tube, heart and placental defects, which persisted for five generations, through transgenerational epigenetic inheritance (Padmanabhan et al., 2013). Folates are co-enzymes responsible for the 1-carbon (1C) unit transfer important for purine, pyrimidine and methionin synthesis, and glycine and serine metabolism (Fowler, 2001). The methionine synthesized from homocysteine in the folate cycle is the only significant source (in most cells) of the universal methyl donor, *S*-adenosyl methionine (SAM), and is essential for methylation processes (Stover, 2009). Although folates are recycled in the folate cycle, it is essential to accumulate as folates are degraded. Folates, where 5-methyl-THF is the predominant bioavailable form in mammals, can either be taken up directly from dietary sources or produced by metabolism of dietary folic acid (Kooistra et al., 2013).

Folate is principally taken up into cells by a reduced folate carrier, RFC1, and by two different folate receptors with high affinity for folate, FOLR1 and FOLR2 (Kooistra et al., 2013). Other members of the receptor family are FOLR3 and FOLR4. The folate receptors are attached to the extracellular side of the membrane by a glycosylphosphatidylinositol anchor (GPI-anchor). The folate receptors are heavily glycosylated and bind folic acid and 5-methyl-tetrahydrofolate (5-methyl-THF) with high affinity (Brigle et al., 1994; Chen et al., 2013). RFC1 has been reported expressed in cumulus-oocyte-complexes (COC) but not in preimplantation embryos, suggesting a role in folate accumulation in the COC cells, but without transport into the enclosed oocyte (Kooistra et al., 2013). FOLR1 has been shown to be an important receptor for the uptake of folates into the cells and has been found expressed in mouse preimplantation embryos, increasing from the 2-cell stage onwards (Kooistra et al., 2013). FOLR2 has not been reported expressed in neither COC nor preimplantation embryos (Kooistra et al., 2013) and binds folic acid with lower affinity than FOLR1 (Brigle et al., 1994). FOLR3 does bind folic acid, but it is a constitutively secreted protein rather than a membrane bound one, probably due to the lack of an efficient signal for glycosylphosphatidylinisotol (GPI)-anchor attachment (Shen et al., 1995). FOLR4 (Juno (FR-d in humans)) is not a typical member of the Folate Receptor Family, but share a high structural homology with FOLR1 (Spiegelstein et al., 2000). Juno-deficient mice are infertile (Bianchi et al., 2014).

The basis of folate supplementation of folate has been administrated as oral or intravenous folinic acid (5-formyltetrahydrofolate) treatments that has been shown to improve clinical status. Folinic acid can be transported through the RFC and participate in folate dependent reactions compared to both folic acid and MTHF that needs further processing (Desai et al., 2016; Frye et al., 2016; Ramaekers et al., 2016).

Mice deficient of *Folr1* are defective in early embryonic development (Piedrahita et al., 1999), in contrast to *Folr2*-deficient mice that developed normally (Piedrahita et al., 1999). *Folr1*^−/−^ embryos died at day E10 with severe morphological abnormalities, and interestingly, these phenotypes could be reverted by maternal supplement of *Folr*^+/−^ dams with folic acid (Piedrahita et al., 1999). The *curly tail* (*ct*) mouse provides a model for neuronal tube defects (spina bifida and exencephaly) (Van Straaten and Copp, 2001). However, folate supplement does not prevent NTD in *ct/ct* embryos (Burren et al., 2010), but formate supplementation enhanced the folate-dependent nucleotide biosynthesis and prevented spina bifida (Sudiwala et al., 2016). Interestingly, primary cultures of mouse embryonic fibroblasts established from *Folr1*^−/−^ embryos revealed altered signal transduction in pathways including transforming growth factor beta 1 (TGFβ1) and the canonical Wnt signaling pathways suggesting that pathways for proper development are significantly altered (Warner et al., 2011).

Besides a role in signal transduction, it has been shown that FOLR1 can act as a transcription factor. In cell lines, FOLR1 translocated to the nucleus and interacted with FGFR4 and HES1, and was suggested to regulate a wide range of developmental genes (Boshnjaku et al., 2012).

In this study, we noted the presence of maternally contributed FOLR1 during mouse and human ovarian follicle development. In mouse follicles, the presence of FOLR1 was observed in both oocytes and the surrounding granulosa cells. In contrast, FOLR1 was restricted to oocytes in follicles from human tissue. In 2-cell embryos, FOLR1-eGFP fusion protein was detected as cytoplasmic and membrane-associated dense structures, resembling the observed cellular localization in ovarian follicle development.

Microinjections of short interference (si)RNA probes targeting the *Folr1* transcript revealed an efficient knock-down of the embryonic *Folr1* transcript. Interestingly, siRNA-mediated knock-down of *Folr1* in zygotes reduced the ability of embryos to develop to blastocyst. This indicates that maternally contributed FORLR1 protein and zygotically synthesized *Folr1* transcripts sustain embryonic development combined.

## Results

### *Folr1* transcript is detectable from 2-cell stage onwards during mouse preimplanation development

**Figure 1:**
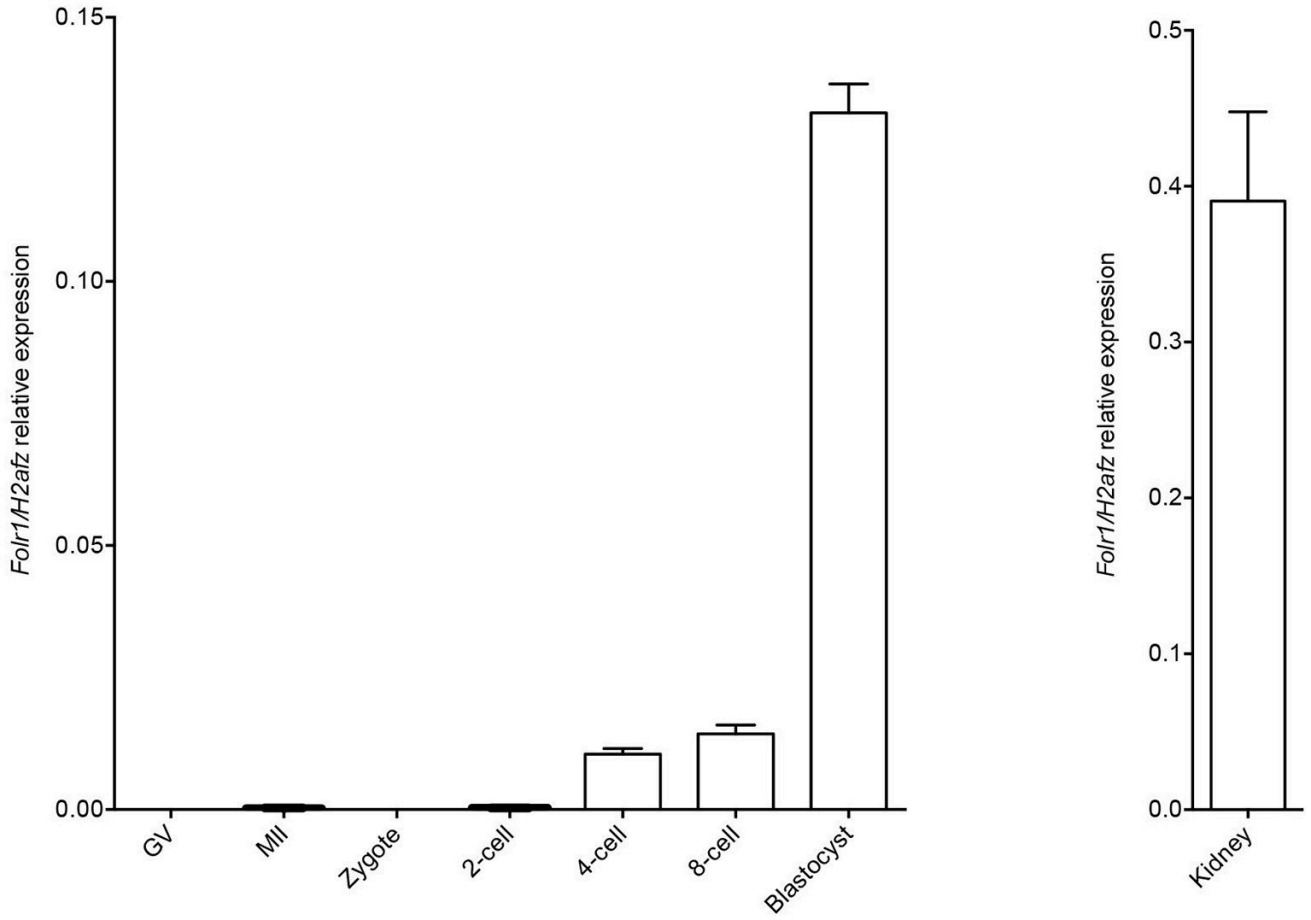
*Folr1* gene expression in oocytes and preimplantation embryos. *Folr1* expression and relative abundan in GV and MII oocytes and preimplantation embryos, as indicated. Kidney tissue was included as a positive control. *Folr1* expression levels were normalized by *H2afz* and relative expression displayed. Data are presented as mean standard deviation SD (bars) of triplicate measurements including standard deviations.

We first wished to analyse if the *Folr1* transcript would be expressed in germinal vesicle (GV) and metaphase II (MII) oocytes as well as during preimplantation development, and thus, if the transcript would be expressed solely from the zygotic genome. Toward this end, RT-qPCR was performed to analyse the expression pattern of *Folr1* during preimplantation stages of embryonic development (Figure 1). Histone *H2a.z* mRNA was used as the most stable internal reference gene during preimplanation development (Jeong et al., 2005; Albertsen et al., 2010). The expected size of *Folr1* and *H2af.z* PCR products were verified by gel electrohoresis (data not shown). The qPCR analysis revealed that *Folr1* was detectable at very low expression at the 2-cell stage, and then its transcript gradually increased to the blastocyst stages (Figure 1). We observed no detecable levels of *Folr1* in GV and MII oocytes (Figure 1). We used tissue from kidney to successfully verify the primer efficiency Figure 1.

This suggests that the *Folr1* transcription is initiated during the first wave of genomic transcripton activation during the 2-cell stage, and remains expressed during preimplantation development.

### FOLR1 in mouse ovarian tissue

**Figure 2:**
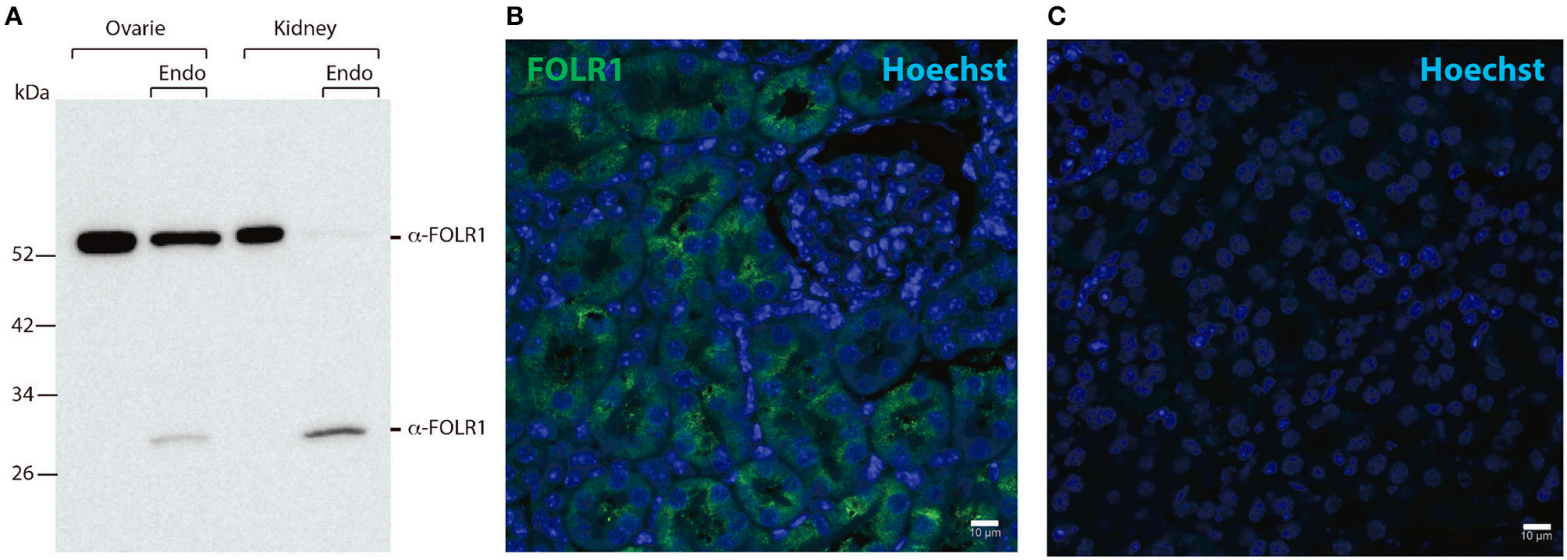
Western blotting and immunohistochemistry controls. **(A)** Western blot of ovary and kidney lysates [with and without Endoglycosidase H (Endo) treatment] from adult mice with antibodies against FOLR1. **(B)** Immunofluorescence staining of kidney from adult mice using antibodies against the FOLR1 (green) **(C)** or without antibody against FOLR1, counterstained with Hoechst (blue) for nuclear stain. Scale bars: 10 μm.

As we did not detect any *Folr1* transcript before the 2-cell stage, we asked if there might be a maternal supply of FOLR1 protein during folliculogenesis. We set out to interrogate the presence and distribution of maternal FOLR1 in mouse ovarian tissue by immunohistochemistry (IHC). Firstly, Western blot analysis was performed to confirm the specificity of the FOLR1 antibody. Western blotting revealed a single protein band of approximately 50 kDa in both ovarian tissue and the kidney control sample (Figure 2A). FOLR1 was estimated to provide a band of approximately 30 kDa, indicating that post-translational modifications such as glycosylation might be added to the receptor. To verify this, the samples were treated with the Endoglycosidase H, a recombinant glycosidase that cleaves within the chitobiose core of high mannose and some hybrid oligosaccharides from *N*-linked glycoproteins. After endoglycosidase H treatment, FOLR1 was deglycosylated and a protein band of 30 kDa was observed in ovarie and kidney lysates (Figure 2A). The ability of the FOLR1 antibody to specifically detect FOLR1 in paraffin-embedded was verified on a positive kidney control (Figure 2B). A negative control (no primary antibody) was included and revealed no detectable staining (Figure 2C).

**Figure 3:**
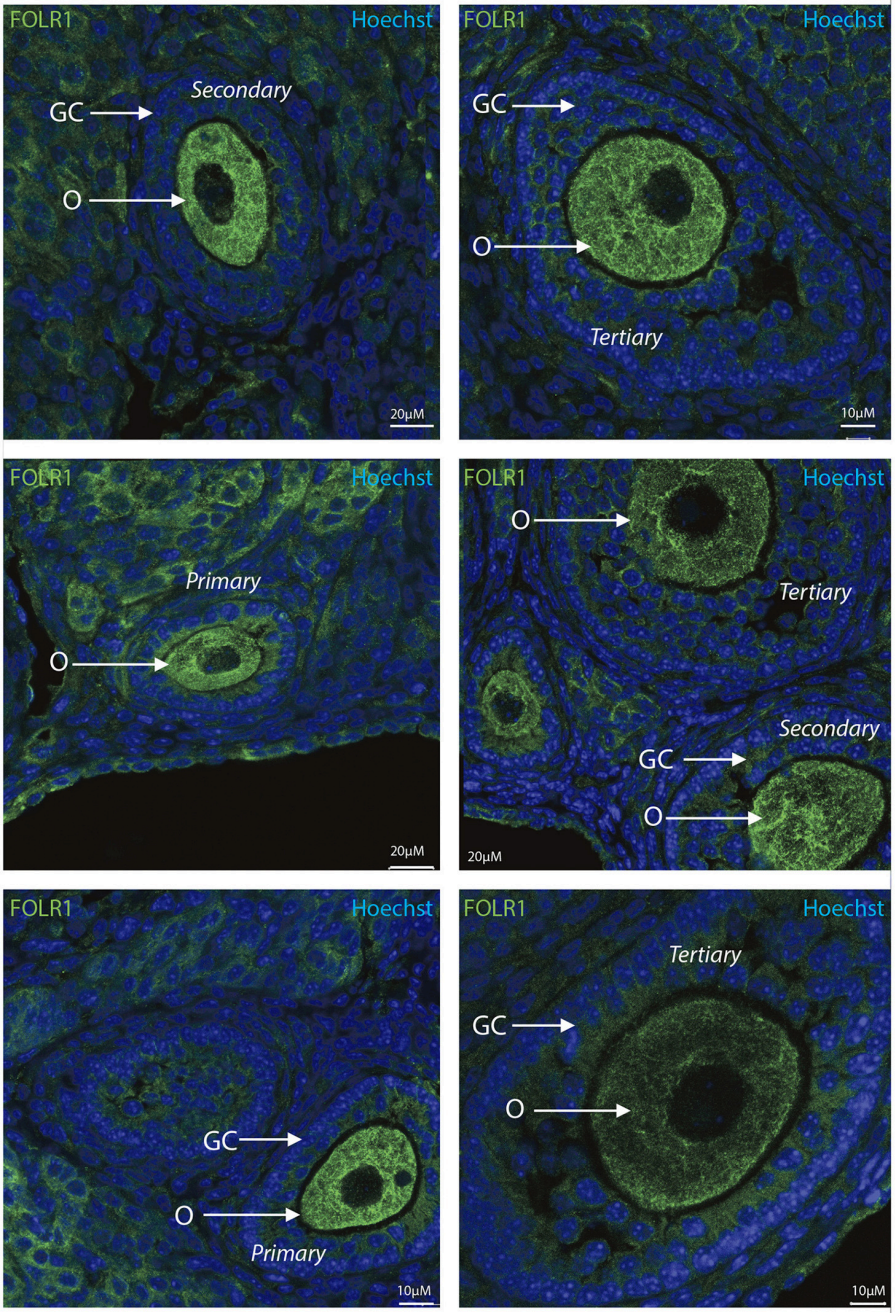
Intraovarian distribution of FOLR in mouse ovary tissue. FOLR1 localized to oocytes in primary, secondary and tertiary follicles. Hoechts staining identifies the nucleus of cells in the slides and the surrounding granulosa cells. o, oocytes; GC, granulosa cells. Scale bars; 10 or 20 μm, as indicated.

**Figure 4:**
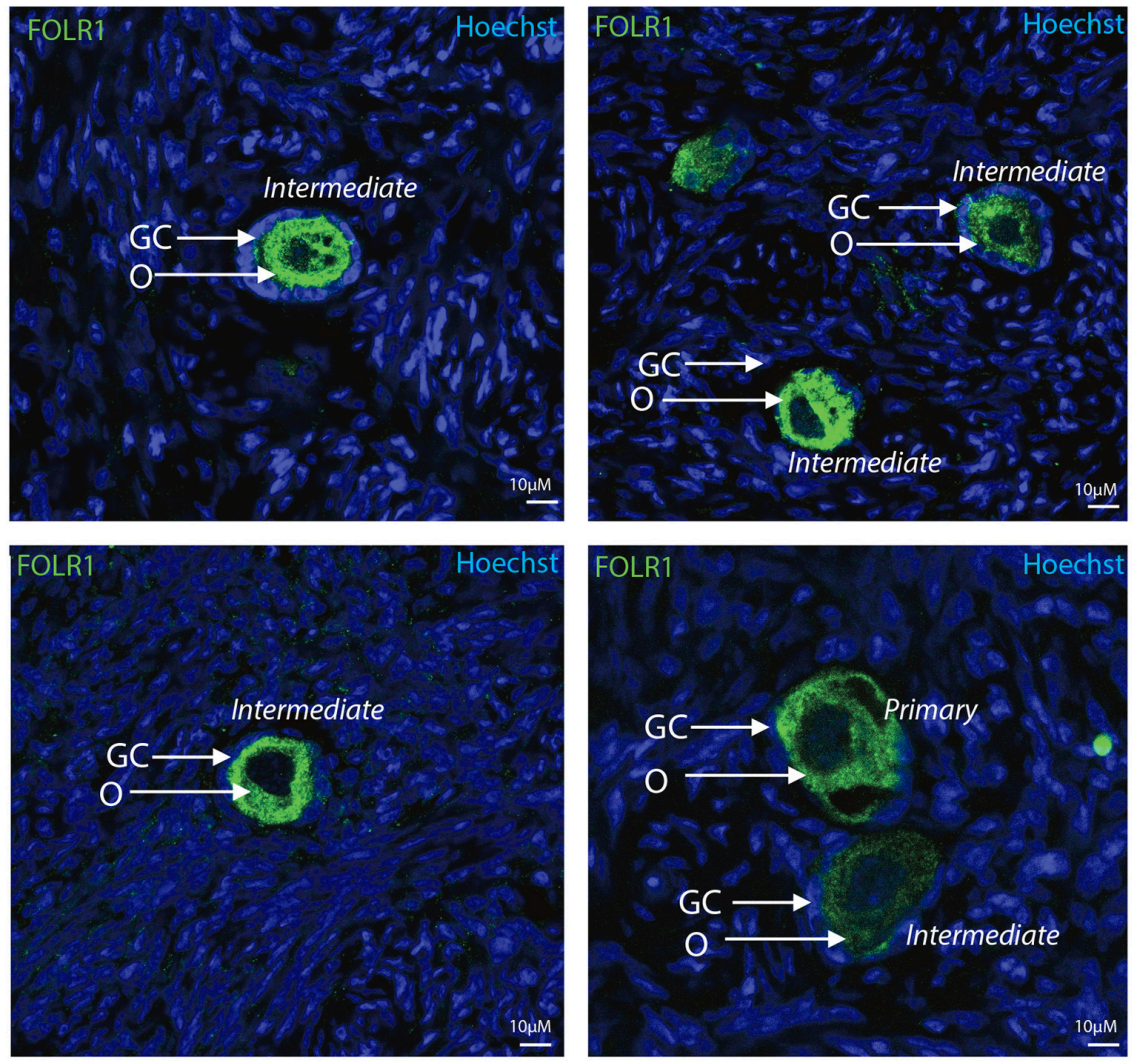
Intraovarian distribution of FOLR in human ovary tissue. FOLR1 localized to oocytes in primary and intermediate follicles. Hoechts staining identifies the nucleus of cells in the slides and the surrounding granulosa cells. o, oocytes; GC, granulosa cells. Scale bars; 10 μm, as indicated.

To determine the presence and intraovarian distribution of FOLR1 in the ovary, IHC was performed on paraffin-embedded and sectioned tissue. In the mouse ovary, the FOLR1 was observed in both oocytes and the surrounding granulosa cells in the tested stages of follicles (Figure 3), indicating that FOLR1 transport is possible in both germ and somatic cells. In human, the FOLR1 was strongly noted in the oocytes of the included follicle stages (Figure 4), and in contrast to the FOLR1 distribution in mice, no FOLR1 was detected in the granulosa cells.

In summary, a pronounced FOLR1 localization was observed in the periphery of the cells compared to the cytoplasm.

### FOLR1 distribution in mouse 2-cell embryos

**Figure 5:**
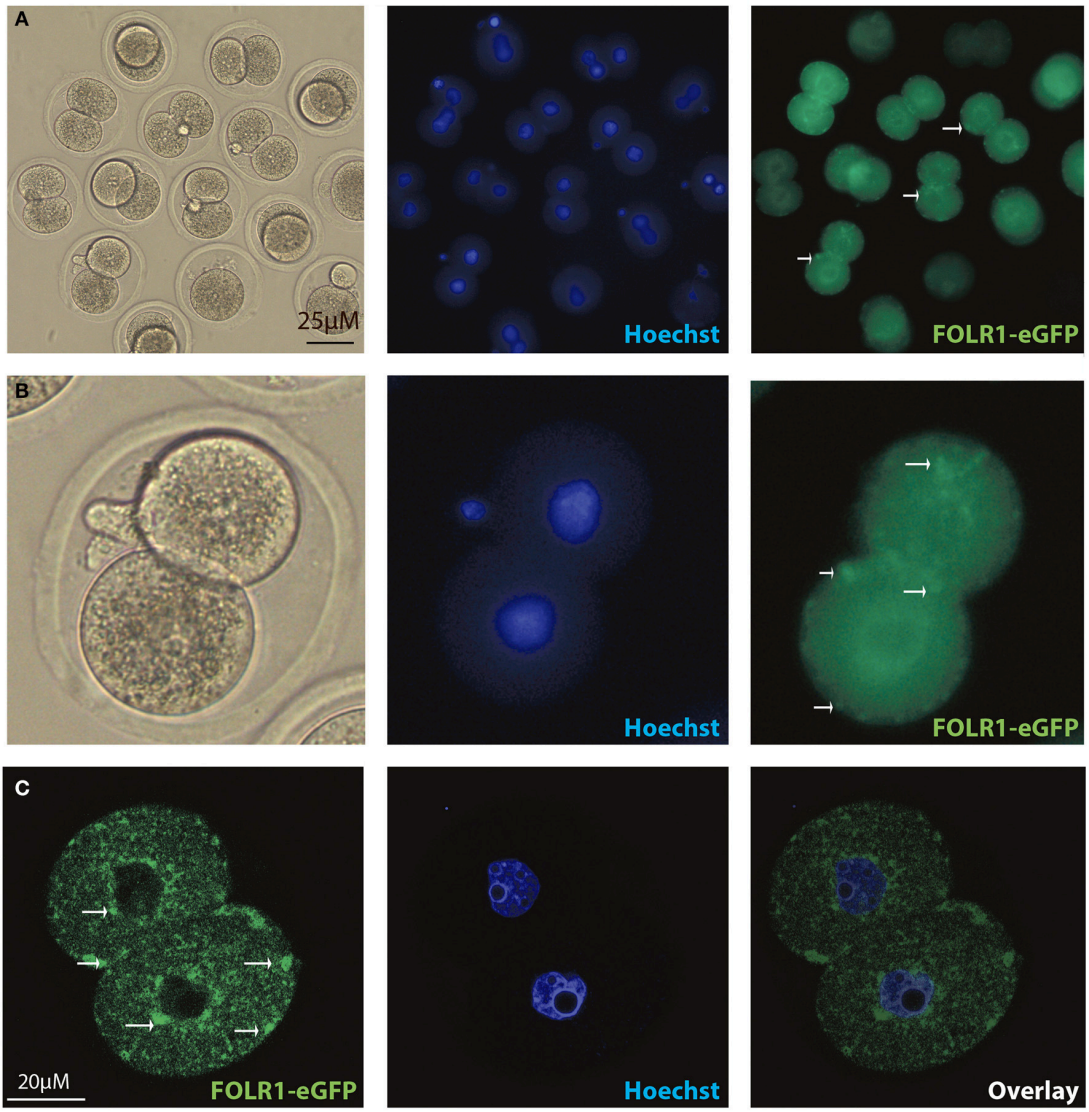
Intracellular distribution of FOLR1-eGFP in mouse 2-cell embryos. Intracelluar immunofluorescent localization and distribution of FOLR1-eGFP in 2-cell mouse embryos using microscopic **(A,B)** confocal imaging **(C)**. Scale bars 20 or 25 μM, as indicated.

**Figure 6:**
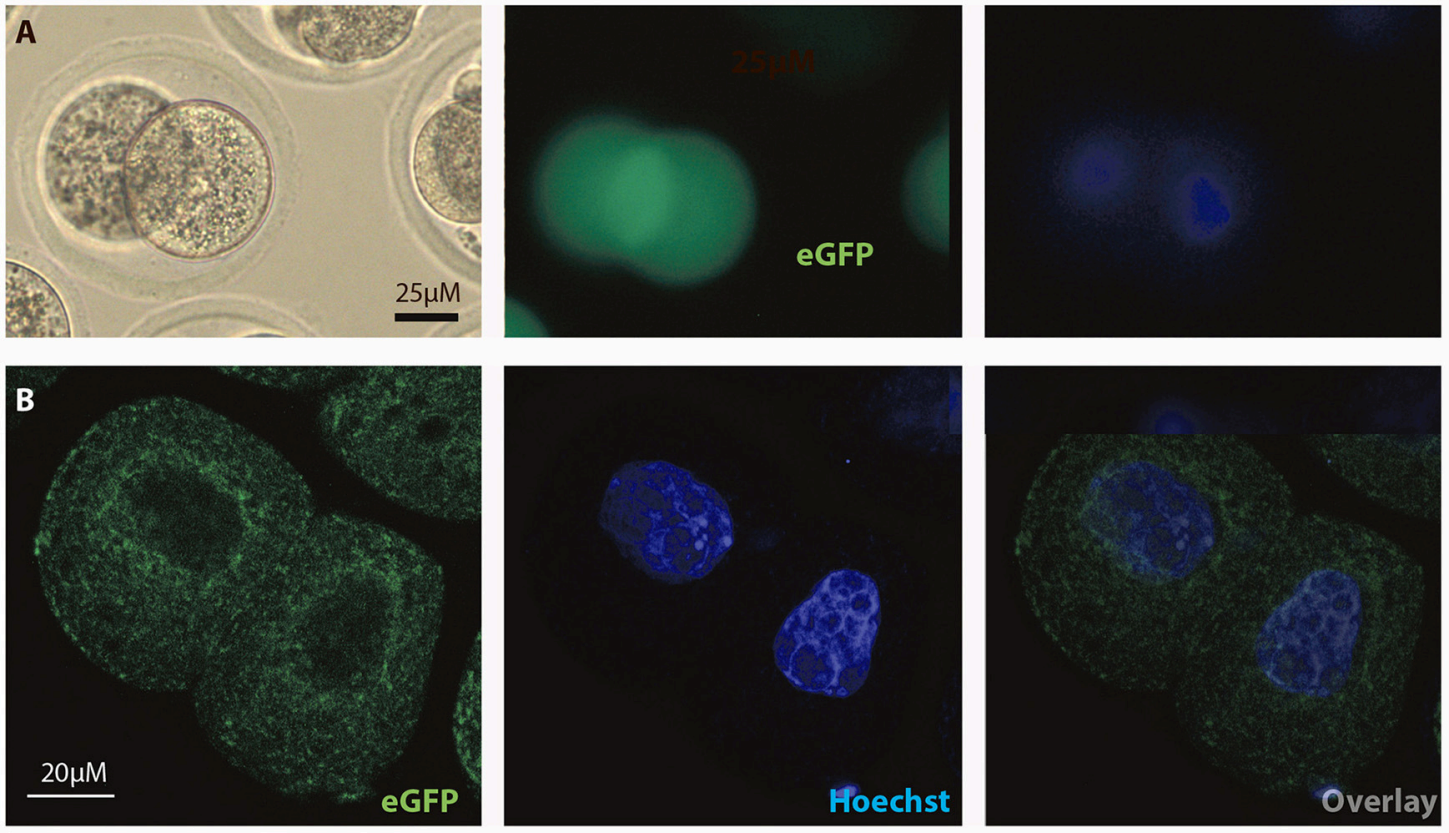
Intracellular distribution of eGFP in mouse 2-cell embryos. Intracelluar immunofluorescent localization and distribution of eGFP in 2-cell mouse embryos using **(A)** microscopic and **(B)** confocal imaging. Scale bars 20 or 25 μM, as indicated.

In order to test if the FOLR1 would display a dynamic intracellular distribution upon fertilization, FOLR1 was fused to enhanced green fluorescent protein (eGFP), to enable us to follow its dynamics and distribution. However, no dynamics was observed in the distribution of FOLR1 between zygotes and 2-cell stage embryos (data not shown). The FOLR1-EGFP distributed in vesicle-like structures in the periphery of the cell membrane, as well as in the cytoplasm (Figure 5). As a control, eGFP localization was observed in the 2-cell embryo, which revealed no specific distribution (Figures 6A,B). In the 2-cell stage (as in zygotes), the FOLR1-eGFP fusion protein resembled that observed for FOLR1 IHC in oocytes and granulosa cells in the ovaries.

### siRNA-mediated knockdown of *Folr1* compromised early developmental potential

**Figure 7:**
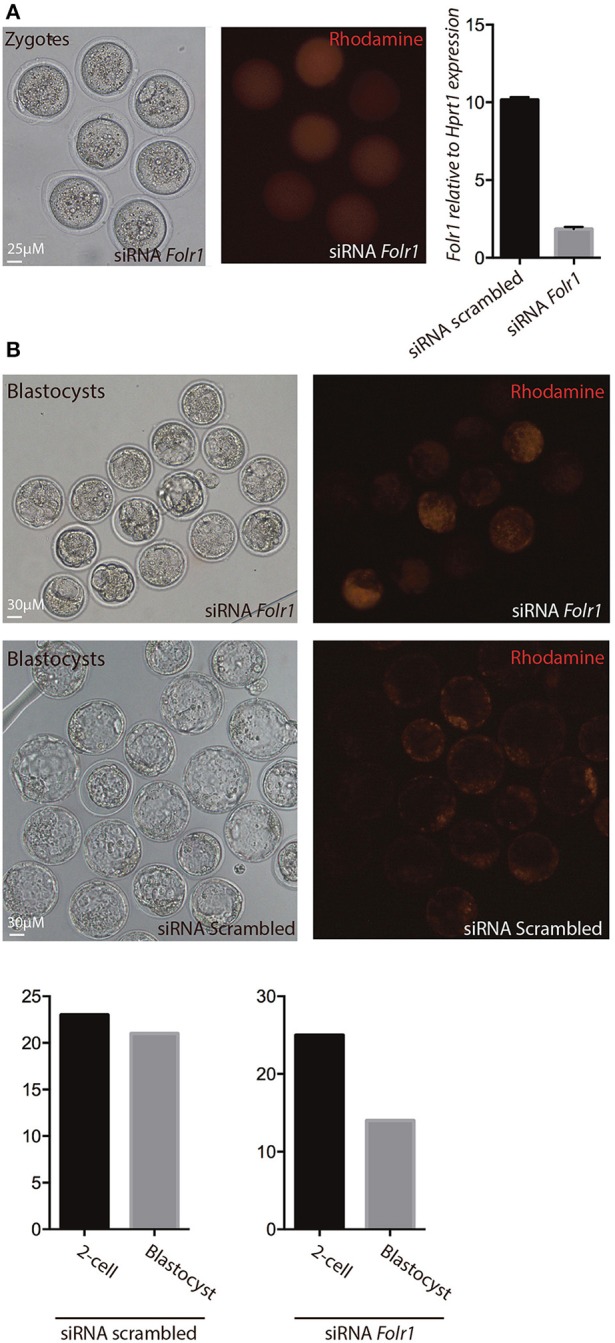
siRNA-mediated knock-down of *Folr1* in mouse zygotes. **(A)** siRNA probes targeting Folr1 (or scrambled control (not shown) was microinjected in combination with rhodamine-conjugated dextrane, in order to identify injected embryos. At the 2-cell stage, qPCR showed that *Folr1* transcript is efficiently reduced after *Folr1* RNAi. **(B)** After *in vitro* culture, siRNA-injected embryos (both RNAi scrambled and *Folr1*) developed into blastocyst, however the capacity to form blastocysts was reduced in embryos that received siRNA targeting *Folr1* (56%) compared to the control RNAi group (92%). Scale bars 25 or 30 μM, as indicated.

In order to reveal whether or not newly generated zygotic *Folr1* transcripts would be necessary to support early development (along side the maternally contributed FOLR1 protein), RNA interference (RNAi) was used as an approach to knock-down *Folr1* transcript. SiRNA-mediated knock-down of the *Folr1* transcript was accomplished by microinjection into mouse pronuclear zygotes. SiRNA probes targeting *Folr1* or scrambled (non-targeting) RNA (co-microinjected with rhodamine-conjugated dextran into zygotes in order to identify embryos that has received siRNAs) were microinjected into zygotes at the pronuclear stage (three independent experiments) (Figure 7A). After an overnight *in vitro* incubation, 2-cell stage embryos were collected for qPCR analysis, which revealed that the *Folr1* transcript was effectively reduced in *Folr1* siRNA-microinjected embryos (Figure 7A).

The embryos were subsequently monitored for their ability to develop into blastocysts. In the group of embryos injected with siRNA scrambled, 92% (21 out of 23) of the 2-cell stage embryos developed into blastocyst, where in the group that received siRNA targeting *Folr1*, only 56% (14 out of 25) of the 2-cell stage embryos developed into blastocysts Figure 7B.

This indicates that zygotic *Folr1* is indeed required to foster development, however, maternally contributed FOLR1 protein can sustain embryonic development. It might be that this is a fine-tuned gradient and dependent on the level of maternal FOLR1 protein contributed.

## Discussion

Dysfunctional maternal folate metabolism caused by folate deficiency or polymorphisms are associated with congenital abnormalities, intrauterine growth restrictions, placental and cardiovascular abnormalities as well as neural tube defects in human. While parts of the abnormalities can be directly associated with the embryonic expression of FOLR1, it is likely that maternally contributed FOLR1 might already mark ovarian cells before fertilization, and influence pre- and post-implantation development.

Transcripts encoding the FOLR1 has not been investigated in ovarian follicles. However, a recent study enterrogated the transcriptomes in human oocytes from primordial and primary follicles, which did not detect expression of the *FOLR1* gene (Ernst et al., 2017). Whether granulosa cells from primordial and primary follicles express the *Folr1* transcript remains to be investigated.

In agreements with another study, we found that *Folr1* transcription appeared to initiate at the 2-cell stage, which is further emphasized in a study that interrogated amantidine-sensitive transcrips, which included *Folr1* (Zeng and Schultz, 2005). Interestingly, this appears to be conserved between different mice strains, as experiments were performed independently on three different strains [CF1 (Crl:CF1) females (Charles River) and BDF1 (B6D2F1/Crl) males (Charles River) (Kooistra et al., 2013), superovulated female CF-1 mice (Harlan) mated to B6D2F1/J males (Jackson Laboratory) (Zeng and Schultz, 2005) and F1 [CBA (C57BL/6jxCBA) females and males (Janvier, this study).

Secreted FOLR1 proteins are either anchored to membranes via a glycosyl-phosphatidylinositol linkage or exist in a soluble form. The receptor-ligand complex is endocytosed into cytoplasmic vesicles and then recycled to the cell membrane. In our IHC, we accordingly observed FOLR1 at the cell membrane, but also noted it in the cytoplasm, resembling the intracellular transport of FOLR1. This might simply reflect the staus quo of the cell in the absence of active folate transport. Thus, in conclusion, it appears that oocyte folate accumulation must occur during follicle development, where folate is maternally supplied. The observation of the folate receptor in both oocytes and granulosa cells in the mouse follicles, but limited to oocytes in the follicles from human samples remains to be further investigated. Very low levels of the folate receptor and/or specific folate supplement might represent likely reasons that the folate receptor was not detected in human granulosa cells. FOLR1 appeared to be absent from the nucleus during follicle development, which suggest that during these stages in development, FOLR1 does not act as a transcription factor. This suggests that the function of FOLR1 during these stages is perhaps assigned predominantly to folate metabolism and signal transduction. Indeed, the role of TGFβ1 in ovarian physiology is well-know, and due to the fact that folate deficiency can alter this pathway, it might be that FOLR1 could influence this, as well, in ovarian follicles.

The FOLR1 distribution in 2-cell stage embryos was interogated using a eGFP fusion approach and the intracellular distribution of FOLR1-eGFP was comparable to that observed in the follicles. FOLR1-eGFP was concentrated at the cell membrane and further noted in cytoplasm, indicating that the FOLR1-eGFP follows the endogoues folate transport and makes this construct suitable for future studies on folate transport in embryonic cells.

Interestingly, while some of the embryos that were submitted to *Folr1* RNAi were indeed able to complete development to the blastocyst stage, several arrest development during later preimplantation stages. This indicates that during preimplantation development, the levels of *Folr1* transcript and FOLR1 protein are finely balanced to rely on maternally contributed proteins and the generation of zygotically-derived *Folr1*. Thus, this suggests that indeed the maternal supply of FOLR1 protein remain during preimplantation and is partly sufficient to support the development until blastocyst stage. Developmental competences might therefore be much more dependent on the maternal internal pool of folate than previously anticipated and variations might account for folate-associated deficiencies.

In this context, it is curious to note that most *in vitro*-based experiments today are performed without folate supplement to the media, even though the long term consequences of folate deficiency in the medium is unknown. It opens a question whether or not we should in fact supplement *in vitro* culture media with folate. Further studies are needed to address whether variations in folate storage might influence developmental potential accordingly.

The long-term effect of *Folr1* RNAi is not known, and it might be that depletion of *Folr1* could interfere with post-implantation. This is indeed in line with the fact that even low blood folate levels in pregnant women are correlated with higher risk of neural tube deficiency in their offspring (Smithells et al., 1976; Detrait et al., 2005), and periconceptional supplementation of folate reduces the occurrence of neural tube defects (Centers for Disease Control, 1991a,b; MRC Vitamin Study Research Group, 1991). Future studies are needed to elucidate the cellular and molecular mechanisms underlying folate and FOLR1 functions and correlation between FOLR1 in preimplantation and early post-implantation development. This could include the CRISP-Cas9 technology and/or a conditional knock-out strategy to functionally knock-down the folate receptor before fertilization.

## Materials and methods

### Embryo isolation

Embryo recovery and isolation F1 (C57BL/6xCBA) females were injected with 5 IU pregnant mare's serum gonadotrophin (PMSG; Folligon, Intervet) and with 5 IU human chorionic gonadotrophin (hCG; Chorulon; Intervet) 48 and 24 h later to induce ovulation, respectively, as described previously (Hogan et al., 1994), and mated with F1 (C57BL/6xCBA) males. Zygotes, 2-cell, 4-cell, and 8-cell embryos were collected at 26, 46, 56, and 64 h post hCG (Sigma), respectively. Blastocysts were collected at 72 h post hCG (Sigma). Zygotes for RNA injections were collected from female mice 25 h post-hCG. All procedures were approved by the Ethics Committee for the use of laboratory animals in Aarhus University (2015-15-0201-00800 to KLH). Zygotes were collected from the oviducts and treated with hyaluronidase (Sigma Aldrich)(50 μl of a 3 mg/ml solution in 250 μl M2 medium) to remove surrounding cumulus cells and cultured in M2 media (EmbryoMax®M2 medium with phenol red, Specialty Media, Millipore MR-015P-5F) at 37°C. Embryos were cultured overnight in drops of potassium simplex optimized medium (KSOM) (EmbryoMax KSOM Powdered Media Kit, Specialty Media, Millipore MR-020P-5F) supplemented with amino acids and 4 mg/mL BSA (Millipore) (no folate supplementation), under embryo-tested paraffin oil in an atmosphere of 5% CO_2_ in air at 37°C.

### RNA isolation

Total RNA extraction was performed using the RNeasy® Mini Kit (Qiagen). For each stage, 10–20 oocytes or embryos were collected and 350 μl lysis buffer was used. Elution was performed with RNase-free water (Qiagen). For all RNA extractions, a DNase digestion step was performed, using the RNase-Free DNase Set (Qiagen). RNA was subsequently stored at −80°C.

### cDNA synthesis

Ovation® PicoSL WTA System V2 (Nugen) protocol was used to generate cDNA. For each cDNA synthesis reaction, 1.5–10 ng RNA was used.

### qPCR

A qPCR analysis of the expression of the *Folr1* gene was made from GV and MII oocytes, 2-cell, 4-cell, 8-cell and blastocyst embryos using the TaqMan® Gene Expression Assay (Applied Biosystems). Reactions were set up for the *Folr1* gene and for the reference gene *H2afz* (Mamo et al., 2007). The reactions were run on a LightCycler® 96 (Roche) using LightCycler® 480 Probes Master (Roche) (Program: 50°C for 2 s, 95°C for 10 min, 45 cycles of 95°C for 15 s followed by 60°C for 60 s and finally 1 cycle of 40°C for 30 s). All reactions were done in triplets with 100 ng of template cDNA in a total volume of 10 μl containing 2 μl H_2_O, 0.5 μl TaqMan Gene Expression Assays (Applied Biosystems) (see appendix 2), 5 μl Probes Master (Roche) and 2.5 μl template cDNA (40 ng/μl). Kidney was used as a positive control in the qPCR reaction. Experiments were repeated at least three times. Triplicate expression values of each gene was set relative to the reference gene via the ΔΔC_T_ methods (Schmittgen and Livak, 2008). As a negative control, cDNA from no template RT PCR reactions was used. All qPCR data was analyzed using Prism 6, version 6.0 (GraphPad Software Inc., CA, U.S.A.). Data are represented as mean ± SD.

### Cloning of *Folr1*-e*Gfp*

*Folr1* inserts were generated with SuperScript III One-Step RT-PCR System with Platinum Taq High Fidelity (Invitrogen) using kidney RNA as template, and the following primers: Folr1-Xma-F: 5′-NNNCCCGGGatggctcacctgatgactgtgc-3′ and Folr1-Xma-R: 5′-NNNCCCGGGGCTGATCACCCAGAGCAGCA-3′. Using *Xma*I restriction sites, the PCR-amplified *Folr1* insert was cloned into the *Xma*1-digested and dephosporylated pBS_RN3P-eGFP vector (Lemaire et al., 1995), in frame with e*Gfp*. Insert and orientation was verified by DNA sequencing.

### Immunohistochemistry

#### Tissue collection, paraffin embedding, and sectioning

Mouse ovaries and kidneys were isolated from F1 females and kept overnight in 4% PFA before embedding into paraffin. Human ovarian cortical tissue was procured from patients who underwent unilateral oophorectomy prior to gonadotoxic treatment for a malignant disease (unrelated to any ovarian malignancies). Patients were normo-ovulatory, with normal reproductive hormones, and had not received ovarian stimulation with exogenous gonadotropins. All methods were carried out in accordance with relevant guidelines and regulations, and The Central Denmark Region Committees on Biomedical Research Ethics and the Danish Data Protection Agency approved the study. Written informed consent was obtained from all participants before inclusion. Patients consented to the research conducted. In subjects undergoing oophorectomy, a small pieCe of the ovarian cortex is used for evaluating the ovarian reserve and for research purposes (Danish Scientific Ethical Committee Approval Number: KF 299017 and J/KF/01/170/99) (Schmidt et al., 2003).

Paraffin tissues were cut in sections of 10 μm. Hereafter, the sections were deparaffinized in xylene and rehydrated in graded ethanol series (100, 95, 70%, water) for 5 min in each vessel. The tissue sections were transferred to 0.01 M citrate buffer (Citric acid and sodium citrate) for antigen retrieval and washed in PBS, and incubated with donkey serum (1 ml donkey serum and 4 ml PBS) for 30 min before adding primary FOLR1 antibody (diluted 1:100 (FOLR1 Rabbit anti-Human Polyclonal, LifeSpan BioSciences) overnight at 4°C. The next day the tissues were washed in PBS and then incubated for 1 h at room temperature with the secondary donkey-anti-rabbit antibody [Alexa Fluor® 488 donkey anti-rabbit IgG (Invitrogen)] (dilution of 1:300). In the following washing steps, the tissues were counterstained with Hoechst (Sigma) to stain the nucleus, added to PBS in a dilution of 1:7,500. The tissues were mounted using Fluorescent Mounting Medium (DAKO S3023). Sections were analyzed and confocal images were taken using a LSM780 laser-scanning confocal microscope with 20, 40 and 63xC-Apochromat water/oil immersion objectives (Carl Zeiss, Jena, Germany).

#### Western blot

Ovary, kidney and brain cortex were taken out from mice and lysed in a lysis buffer containing 7 ml Tris-EDTA buffer (10 mM Tris-HCl, 1mM EDTA), 1 tablet of Complete Mini EDTA-free protease inhibitor (Roche) and 1% (70 μl) IGEPAL®CA-630 (Sigma-Aldrich). 30 μg of proteins from ovary, kidney and cortex were used for Western blotting analysis. Primary FOLR1 antibody (see appendix 8) was applied in a concentration of 0.5 μg/ml in the blocking buffer and the membrane was incubated overnight at 4°C. The membrane was washed for 3 × 10 min in PBS-Tween and secondary HRP-conjugated polyclonal swine-anti-rabbit antibody (DAKO S3023) was applied in a dilution of 1:1,000 in blocking buffer and incubated for 1 h at RT. The membrane was incubated in a chemiluminescent detection reagent. [ECLTM Western Blotting Analysis system (GE Healthcare)] and developed for 45 s using a ImageQuant LAS-4000 (GE Healthcare).

Endoglycosidase H (New England Biolabs, P0702S) treatment was performed according to the manufactorers instructions (New England Biolabs protocol).

### RNA synthesis, microinjections, and confocal imaging

Generation of mRNA encoding FOLR1-GFP and GFP and microinjection into zygotes were performed as described (Albertsen et al., 2010). Microinjection of embryos with siRNA (ON_TARGETplus Mouse *Folr1*, Smartpool, L-061448-01-0005 and ON-TARGETplus Non-targeting Pool D-001810-10-05) with the final concentration of 20 μM, together with Rhodamine-conjugated dextran as marker of injection) or mRNA (*Folr1-eGFP*, or *eGfp*, 200–400 ng/μl) was carried out in M2 media (EmbryoMax® M2 medium with phenol red, Specialty Media, Millipore MR-015P-5F) covered in oil on a glass depression slide using a Femtojet micro-injection system (Eppendorf). Embryos were cultured in KSOM (EmbryoMax KSOM Powdered Media Kit, Specialty Media, Millipore MR-020P-5F) under paraffin oil at 37.5°C in air enriched with 5% CO_2_. The microinjected zygotes were incubated overnight (37°C, 5% CO_2_) in KSOM (EmbryoMax KSOM Powdered Media Kit, Specialty Media, Millipore MR-020P-5F). The next day, 2-cell embryos were incubated for 15 min in a 1:7,500 dilution of Hoechst (Sigma) in DPBS (Life Technologies), washed three times in PBS-T and fixed for 15 min in 2% PFA, before mounting (Fluorescent Mounting Media, DAKO S3023). Embryos were analyzed using the fluorescent microscope (Leica DMI400B) and Leica LAS Software.

Confocal images were taken using a LSM800 laser-scanning confocal microscope with 20, 40, and 63xC-Apochromat water/oil immersion objectives NA 1.2 (Carl Zeiss, Jena, Germany). Confocal images were exported to ImageJ for image processing.

## Author contributions

KL conceived the study. TS performed IHC/Western blot experiments. SF performed qPCR analyse. EE contributed with human tissue. MN and KL performed confocal analysis. AH conducted qPCR of siRNA experiments. KL performed siRNA microinjections and wrote the manuscript. All authors approved the final manuscript.

### Conflict of interest statement

The authors declare that the research was conducted in the absence of any commercial or financial relationships that could be construed as a potential conflict of interest.
